# On the Discursive Construction of Social Entrepreneurship in Pitch Situations: The Intertextual Reproduction of Business and Social Discourse by Presenters and Their Audience

**DOI:** 10.1007/s10551-022-05161-7

**Published:** 2022-06-10

**Authors:** Karin Kreutzer

**Affiliations:** Chair of Social Business, Impact Institute, EBS Universität für Wirtschaft und Recht, Rheingaustrasse 1, 65375 Oestrich-Winkel, Hesse Germany

**Keywords:** Social entrepreneurship, Impact investing, Storytelling

## Abstract

This study explores the discourse of social entrepreneurs and their audiences in pitch situations. Adopting a practice perspective on social entrepreneurship, we videotaped 49 pitches by social entrepreneurs at five different events in two incubators in Germany and Switzerland. Our analysis of the start-ups’ pitches and the audience’s questions and comments as well as of interview data elucidates the nuances of social and business discourse that social entrepreneurs and their audiences draw upon. Our analysis shows how many social entrepreneurs mobilize a discursive repertoire that is familiar to their business-oriented audience while others predominantly draw on a social discourse. We identify separating, mixing, and combining as key strategies that allow social entrepreneurs to dance between the two. We discuss how the intertextual reproduction of concepts, objects, and subject positions contains both enabling and constraining elements, which results in an ethical dilemma for social entrepreneurs: Should they re-package their social impact story in a business discourse to connect with their audience?

## Introduction

It is complex for social entrepreneurs to convince potential supporters, resource providers, and investors in a pitch situation since many are not familiar with the concept (Dacin et al., 2011; Parhankangas & Renko, 2017). Social entrepreneurship combines potentially contradicting elements of social and commercial logics in unprecedented ways to address social problems (Battilana & Dorado, 2010) and it creates positive impact for society (Stephan et al., 2016). Previous research has focused on various aspects that influence the process of resource mobilization such as signaling the competences of the founding team (Eisenhardt & Schoonhoven, 1990; Shane & Cable, 2002; Shane & Stuart, 2002; Stuart et al., 1999), the network that the entrepreneur is embedded in (Hall & Hofer, 1993; Higgins & Gulati, 2003; Steier & Greenwood, 1995) and the social competence of the founder (Baron & Markman, 2003). A recent turn toward linguistics (Hjorth & Steyaert, 2004) has called attention to the role of pitching (Clarke et al., 2019) and storytelling (Garud & Giuliani, 2013; Martens et al., 2007) in the entrepreneurial process. In this study, we explore how social entrepreneurs present their venture to the audience in a pitch situation. In doing so, we intend to shed light on the specific dilemmas and tensions that social entrepreneurs face.

Zacharakis et al. (2007) found that early stage investors are to some extent intuitive decision makers. This may apply even more to the context of social entrepreneurship since the decision to support social entrepreneurs could also be a gut decision instead of a purely analytical one (Achleitner et al., 2013; Hehenberger et al., 2019). The existing research on pitches by social entrepreneurs is limited (Clough et al., 2019). Two studies on social ventures that presented on the online micro-lending platform Kiva offer contradictory findings on the question of whether business or social discourse in the pitches tends to convince potential funders (Allison et al., 2015; Moss et al., 2015).

While the nascent field of impact investing and resource mobilization for social entrepreneurship is attracting increasing scholarly attention (Buckland et al., 2013; Hehenberger et al., 2019; Höchstädter & Scheck, 2015; Sparkes & Cowton, 2004), we do not yet fully understand how social entrepreneurs pitch and how resource providers react to those pitches. While there is a presumption that the motivation to support and invest in socially oriented ventures is likely to be an ethical act to serve a larger social cause (Dees, 2012), we know little about how such processes unfold.

Specifically, current research falls short in investigating the dialogic nature of a social entrepreneur’s pitch (Bakhtin, 1986; Langley & Tsoukas, 2010). Texts are often constructed with co-authorship where others are asking questions or giving comments (Ochs, 1997). Intertextuality captures the idea that every utterance is inevitably linked to what has been said before (Bakhtin, 1986). The text of a pitch is thus constructed as a mosaic of quotations since any text is the absorption and transformation of others (Kristeva, 1980). Creating such a mosaic is a process by which different words, phrases, and speech acts are selected and combined when constructing the meaning of a phenomenon such as social entrepreneurship. We presently lack an understanding of how a pitch is connected to macro-discourses and how the audience may intentionally or unintentionally co-author the texts produced (Fairclough, 1992).

Adopting a practice approach to social entrepreneurship (Bruder, 2020; Dey & Steyaert, 2016), we analyzed videotapes of 12 (data collection I) and 37 (data collection II) of pitches by social entrepreneurs (or entrepreneurial teams), their audiences and the interaction between both groups in the subsequent Q&A sessions.

The main contribution of our paper is that it advances our understanding of social entrepreneurial pitching as a dialogic process in which both presenters and their audience draw from larger discourses. This interdiscursive perspective allows us to highlight the power dynamics involved in the construction of social entrepreneurship in pitch situations. Our study offers two key contributions to the literature. First, we offer detailed insights into the discourses social entrepreneurs and their audience mobilize in pitch situations. We highlight how powerful gatekeepers predominantly drew on a business discourse offering a “taken-for-granted” understanding of what social entrepreneurship is which goes largely uncontested. Second, we highlight how aligning their presentations with the powerful business discourse has enabling and constraining effects for social entrepreneurs.

## Pitching in Social Entrepreneurship

### Storytelling and Resource Mobilization in Social Entrepreneurship

Resources such as financial, physical, human, as well as intangible resources (Starr & MacMillan, 1990) are central to venture creation (Clough et al., 2019). Since social entrepreneurship as a concept is new to many providers of resources and does not conform with well-known categories of business or not-for-profit (Hockerts, 2010), founders still have to do significantly more convincing. Social entrepreneurship is defined as individuals and organizations that “use a business logic in a novel and entrepreneurial way to improve the situation of segments of the population that are excluded, marginalized, or suffering and are themselves not capable of changing this situation.” (Saebi et al., 2019, pp. 70–71). Even though the term itself was coined more than two decades ago (Dees, 1998), resource providers have discovered the field only recently (Buckland et al., 2013) and many investors face uncertainty in a fragmented field that still lacks standards and oversight.

Early research on impact investing suggests that often the audience of social entrepreneurial pitches is torn between an ideal–typical charity logic and an investment logic (Moody, 2008; Nicholls, 2010). Hehenberger et al. (2019) show how a dominant “field ideology” has emerged among experts in impact investing, which emphasizes “head” over “heart” decisions, impact measurement over storytelling, tools over needs of beneficiaries, and picking the best (“winners”) over nurturing the social entrepreneurial ecosystem. Despite these significant advances, our knowledge about the pitch process itself as well as the interaction between social entrepreneurs and their audience remains limited. Such an audience may consist of actors as diverse as wealthy private individuals or investment advisors (Paetzold & Busch, 2014; Paetzold et al., 2015), representatives of venture capital funds, institutional asset owners (Wood et al., 2013), supporting organizations, foundations, and other experts in the field of social entrepreneurship.

A recent turn in entrepreneurship research has shifted the focus from entrepreneurial characteristics (MacMillan et al., 1986; Muzyka et al., 1996) to entrepreneurial behaviors (Clarke, 2011; Zott & Huy, 2007). In this research stream of entrepreneurial behavior, a group of authors investigates the role of stories in resource mobilization (Cornelissen et al., 2012; Lounsbury & Glynn, 2001; Martens et al., 2007; O’Connor, 2002). Storytelling has been suggested to be of specific importance for social ventures (Clough et al., 2019). While research on “live pitches” in social entrepreneurship is scarce, evidence from studies on texts of crowdfunding presentations is also “meager” and “inconsistent” (Clough et al., 2019, p. 19). Using a different text analysis dictionary led to contradictory results in two studies on the same crowd-based micro-lending platform (Allison et al., 2015; Moss et al., 2015). Thus, to date we lack a coherent understanding of the discourses used in social entrepreneurial pitch situations. We need to learn more about the discourses social entrepreneurs (and their audience) draw upon in live pitches, which may differ from the presentation of a start-up to “laypersons” on an online crowdfunding platform.

### Pitches in the Entrepreneurial Process

Research on pitches in commercial entrepreneurship found the pitch to be a critical component in the funding vetting process conducted by resource providers. A pitch is often the initial introduction to and presentation of the venture to potential investors and resource providers (Chen et al., 2009; Clark, 2008; Wiltbank, 2005). Resource providers use this forum to evaluate both the potential of the venture and the entrepreneur’s capabilities to lead and grow the venture (Fried & Hisrich, 1994). It provides a critical opportunity for the entrepreneur to articulate the venture’s business propositions to create interest (Hargadon & Douglas, 2001; Mason & Harrison, 1996). If resource providers form negative impressions of the entrepreneur’s abilities to lead the venture during these presentations, it is improbable that access to funding will later be secured (Martens et al., 2007; Mason & Harrison, 1996; Mitteness et al., 2012).

In pitches, connecting with the audience is especially important in the process of mobilizing resources as it helps to frame the nature of the business in such a way that its potential value becomes believable (Aldrich & Fiol, 1994; Lounsbury & Glynn, 2001; Martens et al., 2007; Smith & Anderson, 2004). For example, Allison et al. (2017) found that characterizing the venture as a personal dream in a pitch narrative positively influenced inexperienced, first-time funders. Moderate accounts of narcissistic rhetoric enhance crowdfunding performance; however, the impact of narcissistic rhetoric becomes detrimental to performance when used extensively (Anglin et al., 2018). Drawing on text, speech, and video metadata of crowdfunding campaigns, Kaminski and Hopp (2019) found that linguistic styles that aim to trigger excitement or are aimed at inclusiveness are better predictors of campaign success than firm-level determinants. While we know from existing research about the pivotal role of pitches in the entrepreneurial process (Clarke et al., 2019), “the interactions between entrepreneurs and different resource provider audiences’” in live pitch situations of social entrepreneurs warrants further scholarly attention (Clough et al., 2019, p. 20).

### Intertextuality

To date, it remains unclear how social entrepreneurs and their audience connect the texts that they produce in pitch situations to larger discourses. Work on discursive devices (Whittle & Mueller, 2012), interaction analysis (Schegloff et al., 2002) or psychonarratology (Bortolussi & Dixon, 2003) take readership and response into account and point toward the dialogic nature of texts where “readership and interpretation are as important as structure or authorship” (Barry & Elmes, 1997, p. 431). The notion of intertextuality holds that every utterance is inevitably linked to what has been said before in other texts (Bakhtin, 1986; Fairclough, 1992; Langley & Tsoukas, 2010). All utterances are populated and constituted by snatches of others’ utterances, more or less explicitly (Fairclough, 1992). Besides capturing the idea that each text connects to earlier texts, intertextuality furthermore draws on a social connection between the author and the reader. To communicate effectively, speakers have to draw on “commonplaces” (Bourdieu, 1998): “the more the communicators’ cultural worlds overlap, the more effective their communication is” (Brannen, 2004, p. 599). Intertextuality also highlights the anticipation of reading (Boje, 2001). Consequently, social entrepreneurs are consciously or unconsciously influenced by the audience they address.

The existing research on storytelling in the entrepreneurial process has not sufficiently accounted for intertextuality, that is, the role the audience plays in co-producing texts in pitches. This study aims to expand the existing work by analyzing the discourses that social entrepreneurs and their audience employ in live pitches. Therefore, we ask: *What discursive repertoire do social entrepreneurs and their audience draw upon in pitch situations?*

## Methodology

To analyze the dynamic and interactive processes that occurred between the presenters and audience in real-time, we chose a qualitative research design (Lee, 1999). We studied live events at popular incubators to explore how social entrepreneurs pitch and how the audience reacts to these pitches. Our research was guided by a focus on the “doing” of social entrepreneurs and their audiences in line with previous research that has conceptualized social entrepreneurship not as an inherently ethical concept “per se” but calls to investigate the concrete practices at play (Bruder, 2020; Dey & Steyaert, 2016). According to the entrepreneurship-as-practice literature, those practices encompass the meaning-making, identity-forming and order-producing actions performed by multiple actors in the entrepreneurial process (Chia & Holt, 2006; Sklaveniti & Steyaert, 2020; Thompson et al., 2020). We videotaped the pitches and the subsequent Q&A sessions to capture the multitude of human actions and interactions which unfold so quickly that they would otherwise have been lost (Christianson, 2018; LeBaron et al., 2018). These videos provided a detailed and permanent record of the events from a perspective that captured both the presenters and their audience.

### Data Collection I: Research Setting

We were initially guided by our general interest in pitches by social entrepreneurs. For this reason, we conducted a first study in an incubator in Switzerland in 2013. In that setting, we had the unique opportunity to record live pitches on video and conduct interviews with social entrepreneurs and jury members. The Swiss incubator was founded in 2011 in Zurich and pursued the following mission: “we are committed to creating a thriving innovation ecosystem where people across organizations, cultures, and generations work together to solve the great challenges of our time.” The pitches that we filmed took place at the end of a 6-month training program for social entrepreneurs. The pitches lasted approximately 10–15 min, followed by a 10-min Q&A session with questions from the jury. The jury consisted of three members from the incubator team, among whom was a university professor. This event was a unique opportunity to gain first insights into live pitches and the discourses that the teams drew upon. However, the data collection at this pitch event presented us with a decisive disadvantage: Although the pitches were evaluated by the jury, this evaluation had few to no direct consequences for the founders. For this reason, we approached a large incubator in Germany for an additional data collection and were granted access to the selection process there 2 weeks later. We were thus able to include pitches where the teams were then either given access to resources or not.

### Data Collection I: Sources of Data

In total, we videotaped 12 pitches and the subsequent Q&A sessions. The interaction of the audience with the presenters was especially important for our research design, as it allowed us to investigate co-authorship and the dialogic nature of the production of texts (Bakhtin, 1986).

Additionally, we conducted 15 interviews (with each presenter team and the three jury members) during the pitching event (see Table 1). The interviews with the three jury members lasted about 60 min and were tape-recorded, the informal interviews with each presenter team about 10–15 min. We asked the jury members to explain their written evaluations of the pitches and which parts of the pitch they remembered best. In the short interviews with the presenters, we asked how the teams had prepared for the pitches and why they had decided to present their start-up in the precise manner in which they did. The recorded interviews and the pitches were transcribed verbatim.

The questionnaire that the jury and the other presenters filled out asked them to evaluate the pitch according to the following criteria: comprehensibility, innovation and effectiveness of the idea, expertise and credibility of the founders, financial viability, and feasibility. While the survey did not meet scientific requirements, we were surprised by the results, which showed how negatively the jury and the other presenters (*N* = 31) rated “social impact stories” compared to pitches framed around a business discourse. After finding only partial confirmation and inconclusive explanations of this finding in the interviews, we collected additional data to gain more insights.

### Data Collection II: Research Setting

In our data collection II, we videotaped 37 live pitches in four pitching events at one incubator organization in Germany. The incubator was originally founded in 1994 to guide marginalized young adults in starting up their own business and becoming self-employed. In 2011, they expanded their program and started an incubator for social entrepreneurs. The organization’s mission is to “foster social equality and promotion inclusion through the support of social start-ups.” At the time of data collection, the incubator had 90 employees and offices in eight German cities. To be accepted to the program and receive a scholarship, a start-up must apply and then present their business idea in a pitch.

Each start-up has five minutes to present their idea and seven minutes for discussion with the jury and the audience. All pitching events are public; thus, approximately 20–50 interested individuals attend and also ask questions. However, the majority of questions are asked by members of the jury. The jury decides which start-ups will be awarded the “social impact start” scholarship. Once a start-up is accepted, it can take advantage of several services including the use of a co-working space, coaching and mentoring, legal consulting and access to a network of investors. Taken together, the basic services that the incubator offers to their scholarship holders correspond to a total value of approximately 20,000 Euros.

Each pitching event that we attended consisted of a different composition of jury members; however, two members were part of the jury in all four pitching events observed. Jury members included, for example, representatives from a government-owned German development bank and a leading organization to support social entrepreneurship as well as from commercial start-up incubators. In addition, successful commercial entrepreneurs, a head of CSR from a German car manufacturer, a marketing director of a large firm, the chief digital officer at a multinational IT company, a private impact investor and a journalist from a national newspaper rounded out the jury. A number of jury members either directly invested in start-ups or supported start-ups in the process of resource mobilization while the program was running and beyond. To be invited to the pitching event, the founders submit a short written application that is checked for comprehensiveness and consistency by a team from the incubator. A staff member of the incubator, who is also a member of the jury, conducts a short informal interview with the applicants. However, the other jury members decide without prior information solely based on the pitch and the subsequent Q&A session.

### Data Collection II: Sources of Data

We attended four pitching events, two in Berlin and two in Hamburg, and collected data on 37 social start-ups. All pitches, including the seven-minute follow-up interaction between start-up and jury or audience, were videotaped. Furthermore, we collected all presentation slides and handouts created by the start-ups to support their talk. As a third source of data, we conducted in-depth interviews with three jury members from the incubator, including the organization’s founder, to deepen our initial understanding of the audience’s discourses gained from the question-and-answer sessions of the pitches. The interviews lasted on average 60 min. In those interviews, we asked how a typical pitch unfolded and what elements determined a successful pitch, including examples. We then discussed several pitches with them and asked them to elaborate on why they were accepted into the program or not. We also asked our intervieweees to explain their evaluation of the pitch. In addition, we had informal conversations with social entrepreneurs and jury members right before or after the pitches, during coffee breaks, and after the events.

In the four pitching events that we attended, 37 individuals and teams presented their ideas. Twenty-two were accepted to the program, and 15 were rejected. In each of the four events, the start-up that convinced the highest number of audience members received the “audience award”: “Soccer for Everyone” in Berlin in February, “Fair Handbags” in Hamburg in May, “Urban Bees” in Berlin in June, and “Blindmap” in Hamburg in August (see Table 2 for an overview of the pitches).

In early 2020, 7 years after the pitches, we conducted an internet search to determine whether the start-ups from the second data collection were still alive (Table 2). Eighteen ventures were still active. One venture still existed, but a commercial mission had replaced the social mission. Of the 23 “winners” that had entered the program, 10 were still alive in 2020 (before the Covid-19 crisis hit). Of the 15 rejected start-up teams, 8 were still active—even though they had not received support from the incubator. Interestingly, only two of the four start-ups that had received the “audience award” survived.

### Data Analysis

We transcribed the text of all pitches and the respective Q&A sessions as well as the interviews verbatim, resulting in 370 pages of text. Informed by grounded theory (Glaser & Strauss, 1967), we began our analysis with a fine-grained reading of the data and wrote “story sheets” for each pitch. The videotapes allowed us to watch and re-watch each pitch and subsequent Q&A session numerous times during data analysis. We first coded the text authored by the jury and the audience and the text produced by the pitchers each individually using the coding software MAXQDA. The cross-case analysis of both incubators in Germany and Switzerland allowed us to identify the key themes of audience and pitchers. We aggregated the themes into two broader discourses, namely business and social. We were surprised to discover how the first-order codes of “using a business language” were similar for both groups. Interestingly, though, we found differences in how social entrepreneurs and their audience mobilized the social discourse. For example, our data show how the concepts of beneficiary, fair remuneration, and scaling a solution to a social problem (without immediate profit interests) were almost exclusively used by pitchers.

For each discourse, we identified concepts, objects, and subject positions (Phillips et al., 2004). Concepts are the very means through which social entrepreneurs and their audience make sense of phenomena such as a start-up. They constitute the vocabulary necessary for meaning-making. Discourses not only describe but also construct objects such as a new social venture. Different discourses may produce different objects, thus offering differing and even contradicting conceptualizations of one and the same start-up (Hardy & Phillips, 1999). Discourses also construct subject positions for the actors involved, as they “give the subject different, and possibly contradicting, positions from which to speak” (Jorgensen & Phillips, 2002, p. 17). Such positions determine which social roles and functions as well as their affordances can legitimately be adopted within each discourse. For example, some social entrepreneurs may emerge as business people and others as social changemakers. In mobilizing a specific discourse, powerful actors shape our understanding of social entrepreneurship, which in turn reflects their power to do so (Foucault, 1980; van Dijk, 2008). In this sense, discourses are performative; they accomplish things (Vaara & Tienari, 2002).

We held focus-group-like presentations of our findings with team members from both incubators to clarify our observations. After completing data collection, the first author became a member of the German incubator’s jury to deepen her contextual knowledge on the selection processes. We also used the interview data to challenge our findings, to learn more about the way the presenters prepared for the pitches and about the invisible reactions of the audience to the presentations.

## Findings

Based on our analysis, we highlight the similarities and the differences between the texts produced by social entrepreneurs and their audience in pitch situations. In drawing on prior texts, both groups mobilized elements of larger discourses—albeit in different ways. We show how they “borrow” concepts, objects, and subject positions from a business and social discourse.

### Discourses in Social Entrepreneurial Pitches

We elucidate how pitchers and their audience select bits and pieces from prior texts, giving their language an intertextual dimension, whether or not the speakers intended to do so. Our analysis suggests that in the actual pitch situations, the questions and comments of the audience drew to a large extent on the discursive repertoire of the business discourse. The presentations of most social entrepreneurs, in contrast, involved elements of both the business and the social discourse.

#### The Business Discourse of Social Entrepreneurs and Their Audience

The business discourse is based on a managerialist *ideology* that emphasizes profit-maximizing strategies, efficiency and profit (see Table 3). The main *concepts* of the business discourse include a *unique selling proposition*, *customer*, *pricing*, *marketing,* and *commercial growth.* In addition, the *financial viability of the business model* was a popular concept used by the audience and, to a small extent, some social entrepreneurs. “How are you going to finance that?” was the most frequently asked question during the Q&A sessions. In responding to the question, social entrepreneurs were forced to elaborate on their business model. Some were well equipped to answer those questions by drawing on a business vocabulary; others struggled. A member of the jury explained his reaction to the pitches as follows:

I’m a businessman and - to be honest - I’m looking for people with business acumen who can make things happen. If it cannot be financed, it remains a naive illusion. [observation, pitch event].

In contrast, many social entrepreneurs did not refer to a “business model” at all in their pitches. Those who did emphasized *revenue streams and financing options*. The founder of LoveClothes later explained her choice not to use “business language” as follows:

The term “entrepreneur” was new to me. I have never written a business plan. To be honest, I don’t even know exactly what it is. I studied international labor rights. I live and breathe for the cause and want to empower the seamstresses in India. [interview, social entrepreneur]

Although the pitch was evaluated negatively and there were some critical queries about the financial viability by the jury, the start-up was accepted in the end. It still existed and was operating successfully in 2020.

In contrast, a jury member stressed the importance of *solid business models*:

Between us, there are the tree huggers in social entrepreneurship. They celebrate themselves so much, and you see little progress. We think like business people, and when I speak out in favor of a social enterprise, it’s not because a few naive do-gooders think they have a good idea. The thing has to finance itself. Period. [interview, jury member].

Associated with such business thinking was the concept of a *unique selling proposition* to the paying customer. Social entrepreneurs highlighted the unique features of the product or service and how it *differed from existing offers*: “We see an added value in our offer in contrast to others because the features of the IT solution are unique.” [pitch]. The start-up Medical Backpack, for example, elaborated in-depth on the viability of their business model and their unique selling proposition. They presented their product as more efficient than existing solutions. The audience reacted very positively to this aspect, congratulating the founders on their unique idea: “This is truly a unique product.” [comment, jury member] Directly after the Q&A session, in which many questions focused on the unique selling proposition of Medical Backpack, the next team—before their actual pitch—repeated the same concept: “We differentiate ourselves from other offerings […].”

Interestingly, the audience members used in their questions on the *unique selling proposition* the term “competitor” to refer to other offers: “Who are your competitors?” [audience question]. The notion of “competitor” portrays social entrepreneurs as trying to compete with others, for example by making bigger sales. The term was rarely used by social entrepreneurs, and if at all, in answers to jury questions.

In addition, we identified the notion of “customer” as a key concept in the business discourse:

It is very important to me that they have an idea of what the customer looks like. Who’s buying this and why? The worm must taste good to the fish, not to the angler, or the story won’t pay off. [interview, jury member].

In the presentation by Blindmap, the founders explained how one of the team members belonged to their customer group and had inside knowledge of their needs. The start-up Sustainability Game presented extensive research on their potential customers including a number of informative figures and statistics. Similarly, the team from “Emissionfree Food” stated:

Let’s look at our primary customers. Our customers are adults between 25 and 45 years, they possess a smartphone, and they live in German- or English-speaking countries. This would be 78 million. Out of those 78 million, 80% say climate change is a serious or very serious problem. This means we may have 62 million potential customers. [pitch]

Many start-ups explicitly referred to *customers*, whereas other social entrepreneurs did not address the concept of *customer* at all during their pitch. The pitchers mostly spoke about the *needs and expectations of (potential) customers*; the audiences’ questions revolved around the *number and characteristics of customers* and their *ability to pay*.

Many pitchers also made use of other concepts originating in the business discourse, for example, *marketing*, addressing topics such as how to *present and advertise the product or service*. One team described their sales channels as follows: “We plan to work with classic retailers, and, of course, organic supermarkets are at the top of our list.” [pitch]. One of the jury members also emphasized the importance of marketing targeted toward the paying customer:

They must be able to demonstrate where they want to get the paying customer. How do they plan to advertise and sell the products or services? [interview, jury member].

Unlike the social entrepreneurs’ presentations, the audience questions were often targeted at “sales” or “sales channels.” Some pitchers had difficulty responding.

Furthermore, jury members asked questions about the *pricing* if the concept had not been mentioned in the pitch: “Can you tell us more about the price? […] How does this cover your costs?” The audience repeatedly emphasized the important of a *pricing strategy* and whether it matched the *costs* and the willingness to pay. Pricing was much less referred to by the social entrepreneurs, or only when asked about it.

In addition, the concept of *commercial growth* was frequently referred to in pitches and the Q&A sessions: “You have to identify new markets to be successful.” [comment, jury member]. The start-up Blindmap explained their strategy for revenue growth as follows:

Our next challenge is to gain automation. As M. explained, we are currently doing everything manually. With each successive step, we want to automate everything so that our maps will become more and more affordable. This has a large impact on our business model. Our maps need to be financed. [pitch].

The audience asked about the potential for *growth in revenues and sales* while some social entrepreneurs also mentioned how they planned to *grow the business beyond the core*. The founder of LoveClothes was very skeptical about growth as such:

I don’t want investors who will reduce our impact and only want a quick profit. If anything, we will grow organically: Slowly and steadily. [interview, social entrepreneur]

These *concepts* of the business discourse used by the pitchers and their audience bring an *object* into being, namely the *social start-up as a business*. The social cause that the venture aims to address is *framed as a business opportunity* rather than as a societal challenge: “We saw a market here.” [pitch]. Urban Vegan Farming chose not to speak about the problem but rather focus on the business opportunity:

We observe a trend that hip, urban people want to feel a connection to their food. This is our target group. We offer harvesting stations; we create a world of experience and a meeting place. [pitch].

Instead of describing the various sustainability problems of the food industry, the founders illustrated the untapped market potential that they wanted to address with their offer. Thus, they *presented the opportunity in a social problem*. The jury rated their pitch as very convincing in the questionnaire. In the interview, the presenters explained their choice as follows:

This is supposed to be a business. We need to convince potential investors of our business model and our potential. […] We have learned to stop boring people with our litanies about the scandalous food industry. No one wants to hear that. Some people know that and the others do not want to hear about it. [interview, presenter]

In line with a business ideology of profit-maximizing strategies and an emphasis on efficiency, conceptualizing the start-up as a business also included the conviction that a market-based solution is superior:

There is a significant difference between the work public agencies or nonprofits do. The laws of the market ensure that social enterprises are efficient. [interview, jury member]

The *subject position* as a legitimate position to speak from in the business discourse portrays the social entrepreneur mainly as an entrepreneur, i.e., as *a person who takes risks and makes money:*

This is our co-working space, where our entrepreneurs really take off. For example, Alpha, they just made a six-figure sum with a crowdfunding campaign. [observation, employee from Impact Lab guides the jury members through their office space]

Founders described themselves as a “business person” with “strong implementation skills.” Their audience voiced how social entrepreneurs should be “entrepreneurs” and “self-starters.”

#### Social Discourse of Social Entrepreneurs and Their Audience

In contrast, the social discourse centers on the main goal of the start-up: working toward a social mission. In the Q&A sessions, only a small fraction of the questions by the audience referred to concepts of the social discourse. Social entrepreneurs drew to varying degrees on the language and concepts of this discourse.

Firmly rooted in a social *ideology*, the social discourse is shaped by values such as justice, inclusion, and responsibility and emphasizes *social impact* (see Table 3).

A central *concept* is the notion of the *theory of change* and *social impact,* that is, how and why a desired social change is expected to happen. To our surprise, only a few questions of the jury referred to the concept and if so, then only to the *reach of the offer*.

By contrast, many social entrepreneurs explained *the social or environmental problem* they intended to address in detail, e.g., the challenge of improving the lives of disadvantaged groups or fighting climate change: “This is challenging. How can I present such a complex problem in such a short time?” [pitch]. The description and analysis of *the problem* ranged from stories the social entrepreneurs had experienced to more abstract presentations of facts and figures. The social cause is framed as a problem that needs to be addressed, emphasizing the drive of the venture for social change. A central part of the pitch was describing the *solution* idea for the problem, i.e., how the founders planned to achieve the intended social impact. The pitchers explained how their innovative idea would bring about social change, promote justice, and responsibility and foster the inclusion of beneficiaries.

For example, the founder of Children’s Bus explained her theory of change as follows:

I know, it’s challenging to explain such a complex social problem in 60 seconds, but I’ll try. […] I’d like to introduce you to Dorusch, Vanessa, and Abdul Kabir. They live in this house, they go to school, they have the soccer field, and that’s what their lives look like – every day. […] They say: ‘I am going to do what mom or dad does.’ Or, even worse: ‘I am going to live on social security.’ They are influenced by what they see. […] I have this bus, 75 square meters with a kitchen. I am going to take them on simple day trips. […] And I am going to run a small restaurant on the bus to finance the trips. [pitch]

Also, social entrepreneurs used the notion of value-added for society [“Gesellschaftlicher Mehrwert” in German] to describe the potential impact of a venture:

If we stop hooligans from becoming violent, we save the government money. We contribute to peace and security and show these people a new way. [pitch].

The founders of Children’s Bus, Multi-Generation house, Local Community, and World Music Concerts used their pitch time exclusively drawing on the social discourse and received unfavorable feedback from the jury members, who questioned “the lack of a business model.” However, in a few cases the "theory of change" was explicitly questioned by the audience: “How are you going to work with the kids?” [audience question].

Other *concepts* that formed an inherent part of the social discourse included *beneficiary*, *fair remuneration, reaching beneficiaries*, and *scaling*. In the social discourse, the *concept* of a *beneficiary* was used instead of *customer*. While the term customer in the business discourse is associated with a certain “willingness to pay,” the concept of beneficiary puts *the in-depth knowledge of the needs* of a marginalized population center stage:

Annett is 14 years old and grew up in one of the biggest slums in Kibera (Kenia). Every month when she has her menstruation, she is afraid to ask her father for money because money is always scarce. This leads to Annett not going to school when she has her period for fear of bleeding through her clothes and embarrassing herself. Annett is not an isolated case. Every month, more than a billion women struggle to afford pads. Out of fear of embarrassment, they stay away from school or work. UNICEF and UNDP state that menstruation is the main reason why girls do not go to school regularly. [pitch]

Even though the start-up made it clear that their idea is a menstrual product primarily for use in developing countries to help women in need, the first question from the audience was:

I’d be interested to know, I know this from the US and I think it’s also available in Denmark. What is the growth potential in Germany? [audience question]

In almost all pitches, the beneficiaries were explicitly mentioned, and often presented in detail using figurative language and introducing a specific persona. In the social discourse, the beneficiary is portrayed as a disadvantaged and/or marginalized person who needs (and receives) help and support and who deserves *justice*. In some instances, the terms “client” or “patient” was also used.

In contrast, the characteristics of beneficiaries were almost never a topic during the Q&A sessions. The term itself was not used at all. An incubator staff member commented on our observations as follows:

One aspect that many on the jury pay far too little attention to is that we should be looking for founders who know their stuff. You don’t just turn a jobless migrant into an entrepreneur overnight. That would be naive. Social change does not happen like that. [interview, jury member]

This is interesting since the concept of *beneficiary* could be viewed as less empowering than *customer* (a person with the right to choose rather than the beneficiary of benevolent giving). Thus, the term beneficiary could actually undermine key values of the social discourse ifself such as *inclusion* and *responsibility.*

*Reaching the beneficiaries* was another key concept of the social discourse. In contrast to the *marketing* concept of the business discourse, the notion of reaching the beneficiaries is concerned with the *accessibility of their offer* and ways to *include beneficiaries* to increase the social impact. It was not a frequent topic in the pitches, but the few who referred to *reaching the beneficiaries* described how they planned to tailor their offer to meet the needs of the target group: “Our App will be easy to use. The gamification will allow users to have fun.” [pitch]. The founder of “Multi-Generation house” explained their choice to dress up with an old curtain over her head as the *“*soul of the building which attracts people from all age groups” as follows: “That’s where we come from. We see the spirit of the building. That’s what invites different people.” We got the impression that the founder felt right at home in her costume as the “soul of the building.” Several jury members, however, made disparaging remarks about it (“ridiculous”) and missed “a business perspective.” In the pitch, the team of “Multi-Generation house” had detailed their track record of how they had consistently brought very different people together. However, as with the vast majority of pitches, the topic was not addressed by the audience in the Q&A sessions. One exception was as an audience member who asked after the pitch of Children’s Bus: “Why should the children want to join your activities?” [audience question] to point to *the number of users needed for (social) success*.

Some social entrepreneurs drew on the concept of *fair remuneration* in their pitches (instead of *pricing*)—but it was also never addressed in the Q&A. *A comprehensive pricing model* was concerned with setting a price that would allow for sustainable production and fair wages:

Our price should reflect the true costs. We do not want somebody else (e.g., the victims of water pollution) to pay the price. [pitch]

Finally, the concept of *scaling* was used by pitchers to depict a vision of how the new idea could be replicated by others in different contexts or expanded to other countries to *grow its social impact*: “This project could be implemented in any city” [pitch]. Unlike *commercial growth*, the notion of scaling is about spreading the word about a solution to a social problem without any immediate profit interest. Thus, the “scalability” refers to the possibility to grow and adapt the idea in other contexts to meet greater needs in future, and it is an often-quoted concept used by supporting organizations in social entrepreneurship. However, no audience question referred to the concept.

These concepts from the social discourse bring an *object* into being, namely the *social impact as the key feature of the start-up*, emphasizing the development of fresh ideas that meet social needs more effectively than existing solutions. The view of social entrepreneurship as an ethical concept draws on an ideology which emphasizes *social impact*, *justice*, *inclusion*, and *responsibility*. A jury member described his preference as follows:

To me, financing, marketing, that’s completely uninteresting at the time of the pitch. You can address that, but you don’t have to. The innovation is important. What is important is the social impact. So what is the problem I want to solve? [interview, jury member]

The rest of the audience co-constructed a slightly different picture of a *business with social side effects*:

It’s a great product and I believe there is a market. […] And I think people will benefit. [comment, audience]

The *subject position* as a legitimate position to speak from sees social entrepreneurs as *activists* and *changemakers: *“Our vision is the reintegration of former forced prostitutes back into society with the help of fashion.” [pitch]. In an interview, a social entrepreneur described herself as a “deeply moral person” and as a *social innovator*:

I want to change the way we look at others. Each person has unique talents - and can give something that no one else has to give. Every person has unique talents and abilities. My aim is to value strengths and not to judge people by their weaknesses. [interview, presenter].

The audience emphasized how social entrepreneurs should *bring new ideas to the table*: “What’s new about this approach?” [audience question].

#### Separating, Mixing, and Combining Business and Social Discourse

Most social entrepreneurs included elements of the business and the social discourse in their presentations. Some drew to a large extent on the business, and others put more emphasis on the social discourse. A third group scarcely referred to either of the two discourses. One presenter described her dilemma in preparing for their pitch as follows:

It’s a difficult choice. You just have so little time. We thought it over and rehearsed it very well beforehand. The problem is, which points should we address? I wrote my master’s thesis on [social problem], so I can already say a lot about that, you know? But there is also - let’s say - a certain community here, so... they want to know where the start-up capital comes from and so on. That’s a certain way of thinking, yes. [interview, social entrepreneur]

Our analysis finds that social entrepreneurs use different ways to separate, mix, or combine the business and social discourse in their pitches. The first group of presenters clearly split the “business” and the “social” part of their presentation, as indicated, for example, by the titles on their slides: “The problem,” “The solution,” “Monetization” [slides pitch presentation].

The second group of pitchers mixed the two discourses—even potentially contradicting concepts. For example, a founder spoke about the “target group,” the “beneficiary,” and the “customer” while referring to the same group of people. The start-up “Live your Dreams” ironically picked up elements of a business discourse to explain their social mission: “Our customers: Dreamers and Storytellers.” [slides pitch presentation]. In several instances, we observed such a use of business vocabulary, which resembled the use of buzzwords without a deeper connection to the rest of the text. Paradoxically, one start-up was expressly fighting against “capitalism” as their ideological opponent; however, they told their anti-capitalism story by drawing on business concepts and a managerialist vocabulary, ignoring apparent contradictions.

A third group set out to combine and thus reconcile potentially contradictory elements of both discourses. The start-up “Upcycling Design” explained how their idea integrates both ideologies:

We are split into two things. Idealism meets realism. We started with our company and said we’d make money and do something social. […] We know our customers. […] Our marketing strategy […]. There is a demand for sustainable products, and it is a trend in the market. Our revenue model […] We wish to include our producers and empower them. It is our way to contribute. It’s about self-realization and recognition for the producers. They can generate an additional income and this is how. […] Our offer is unique since there is no competitor that we know of and because we support our producers and educate our customers. […] On the next slide, this is our business model, and we combine it with idealism. [pitch]

In a story that the audience described as “coherent,” the pitchers combined elements of the business discourse (*company, business model, demand, customer, marketing strategy, revenue model, competitor, unique selling proposition*) with elements of the social discourse *(idealism, sustainable products, include, empower, contribute, self-realization, recognition, support producers, educate customers).* Unlike those who mixed the discourses, potential contradictions between the two discourses in this example did not become apparent in the presentation or were turned into mutually reinforcing combinations as, for example, the idea of “sell by educating, educate by selling.” After their pitch, there was an unusually high number of questions, and the Q&A session was extended. All but one of the questions asked by the audience related to the business discourse.

## Discussion

Our analysis advances prior research on (social) entrepreneurial pitching by offering a sorely needed intertextual perspective that highlights the discursive repertoire social entrepreneurs and their audience mobilize. The conventional view would be to see social entrepreneurs who are trying to a convince a jury which in turn is seeking to choose the best. Such a view would be limited because it does not capture how gatekeepers define what a social entrepreneur is (and is not) in that moment. We highlight the nuances of the potentially contradicting discourses that social entrepreneurs choose from and how those connect to the discursive repertoire that their audience uses. Our detailed analysis of 49 videotaped pitches revealed that many social entrepreneurs align their text—intentionally or not—with the audience’s dominant (business) discourse. Others predominantly focused on social impact stories while neglecting the business discourse except when they had to answer questions from the audience.

### Uncertainty and Power in Social Entrepreneurial Pitching

Despite important advances in research and practice (Saebi et al., 2019), the concept of social entrepreneurship is still contested. Actors in the field and investors face uncertainty in light of the different understandings and definitions of the term (Bacq & Janssen, 2011). In the absence of clear selection criteria, the jury members in our study were largely left on their own to collectively make sense of the presentations. Borrowing from what they knew, they asked questions within the realm of the business discourse familiar to them. Some social entrepreneurs attempted to distance themselves from the powerful business discourse and—in drawing primarily on the social discourse—acted as if they were independent from the audience’s judgements. However, the jury members forced those presenters into the discursive space of the business discourse in the Q&A sessions.

In our study, gatekeepers exercised subtle and nuanced forms of power (Fleming & Spicer, 2014; Lukes, 1974) by normalizing our understanding of what social entrepreneurs are supposed to say and do. In focusing their questioning of the pitchers predominantly on business aspects, they offer a “taken-for-granted” understanding of what social entrepreneurship is (and what it is not), which goes largely uncontested; representing the ultimate exercise of power (Hehenberger et al., 2019; Jacobs et al., 2020).

Our study extends the existing research on the behavior, gesture and language of entrepreneurs (Clarke, 2011; Clarke et al., 2019; Zott & Huy, 2007) since it also deals with the language their audience uses. We offer a critical perspective on the role of gatekeepers, who control access to resources or own financial assets and are more familiar with a business discourse. Thus, the business-oriented field ideology of impact investing (Hehenberger et al., 2019) plays an active role in shaping and influencing the fragmented concept of social entrepreneurship (Saebi et al., 2019). In our case, the jury members decide which social enterprises are legitimate and gain access to resources (or not). In such situations, the extent to which the gatekeepers themselves are dependent on social entrepreneurs can readily be forgotten.

Previous research has suggested that the “content of entrepreneurial stories must align with the audience’s interests and normative beliefs” (Lounsbury & Glynn, 2001, p. 550). Bourdieu (1998) calls this alignment of interests “commonplaces,” something the audience and the author have in common. Research in this field has investigated this connection, for example, in the context of journalists who reproduce commonly held ideas and ideologies to gain the attention of their audience (Kuronen et al., 2005) or how entrepreneurs adapt their dress to fit the specific audience they are addressing (Clarke, 2011). Our analysis extends current research since we elucidate the dialogic nature of texts (Bakhtin, 1986; Fairclough, 1992) including pitchers and their audience. We highlight the intertextual reproduction of the business-driven “field ideology” of their audience (Hehenberger et al., 2019) which seemed to be a process through which entrepreneurs could connect and engage on the basis of a common language and shared concepts. For example, in a few instances, social entrepreneurs repeated words or phrases they had heard from the jury just a few minutes earlier. We show how some social entrepreneurs opened a shared space with their audience by mostly mobilizing the business discourse, which was familiar to the jury. Others resisted—intentionally or not—and were rather forced into this space by audience questions.

### The Discursive De-construction of the “Social” in Social Entrepreneurship

The concept of social entrepreneurship emphasizes the creation of positive social impact for society (Mair & Martí, 2006). Existing research has mainly viewed social entrepreneurship as being “at the service of the common good, thus exhibiting a thoroughly synergetic relationship with ethics” (Dey & Steyaert, 2016, p. 2). However, our data suggest that powerful gatekeepers in the social entrepreneurship community construct a more business-dominated view on how to solve complex societal challenges that has important implications for the distribution of resources for start-ups. Interestingly, experts on the social or environmental challenges that the social entrepreneurs wanted to address were absent from the jury. The jury made decisions on ventures working in the field of female empowerment, education, health, or crime prevention without involving professionals in the respective area.

If “success” in the field of social entrepreneurship is constructed primarily in business terms (e.g., number of customers, profit margin, or revenue growth), the original social purpose will be backgrounded. Thus, while social entrepreneurs have been argued not to be inherently moral beings who do the right thing in contrast to the rest (Dey & Steyaert, 2016), the same may apply to their supporters and investors. Therefore, we argue that gatekeepers may need to re-focus their attention on categories of “social success,” e.g., the theory of change, the quality of programs, the satisfaction of beneficiaries, and the social change accomplished (Parkinson & Howorth, 2008). Thus, our study offers a fresh view on the practice of social entrepreneurship as a movement in which gatekeepers and investors may—unintentionally—suppress the social discourse that they intend to support.

Our study points to a specific ethical dilemma for social entrepreneurs themselves: Which discourses should they align their presentation with? Our study elucidates how social entrepreneurs have to juggle the tension between emphasizing one discourse or another (i.e., customer vs. beneficiary) within the tight time limit given. Including both discourses in the presentation resulted in being able to go less in depth. Some social entrepreneurs used the discourses sequentially and separately, others mixed them while ignoring apparent contradicitions, and a third group attempted to hide contradictions or find mutually reinforcing combinations.

Some social entrepreneurs felt uncomfortable with a business discourse they were not familiar with, but were nevertheless maneuvered into that discursive space by the audience in the Q&A session. Thus, the question arises as to the consequences of such a push toward the business discourse. Conformity with the powerful business discourse *enables* the pitching team to disguise potential contradictions and conflicts inherent in many hybrid enterprises (Battilana, 2010; Pache & Santos, 2010). By actively hiding the complexity of hybridity, business stories helped the presenters simplify reality and create convincing cause-and-effect chains. Our findings illustrate how the social impact logic is “repackaged” in business vocabulary (Mauksch, 2017). In business-oriented pitches, serving beneficiaries and aiming for a social-mission-oriented goal were turned into stories of attracting customers and pursuing commercial growth. Such rationalism implies that by applying managerialist practices and an entrepreneurial mindset to social issues, complex realities may appear well-ordered and manageable to the audience (Dey & Steyaert, 2010). The subject position entrepreneur offered the social entrepreneurs a simple, unequivocally legitimate position to speak from. Such “entrepreneurial stories” seemed to have resonated with a business-oriented audience since the discursive repertoire was familiar to them. In contrast, social impact stories enabled presenters to emphasize the problem, their solution to it and their inherent intrinsic motivation, which may appeal more to the emotions and gut feeling of their audience.

On the other hand, the adoption of the available concepts and “ready-made” elements of the business discourse may also *constrain* the social entrepreneurial community in important ways. The business discourse offers only a limited repertoire, and the hiding of the “social” may suppress critical alternative voices and draw an overly optimistic picture of the extent to which business solutions can succeed in solving complex social and environmental problems (Kreutzer & Mauksch, 2014). Only in a few, rare cases did social entrepreneurs succeeded in combining both discourses and apparently seamlessly hid potential contradicitions between the two—as, for example, the start-up “Upcycling Design.”

Our study extends the existing research on the introduction of the market discourse in the social sector (Davis & Kim, 2015; Hwang & Powell, 2009) and its predominance in social entrepreneurship (Cho, 2006; Parkinson & Howorth, 2008), which has detrimental effects for relationships to constituents (Hwang & Powell, 2009). We illustrate how social entrepreneurs who resist attempts to determine who they should be as their way of practicing ethics (Dey & Steyaert, 2016) may not be able to easily connect with powerful business-oriented gatekeepers and investors. If social entrepreneurs—responding to such pressures—try to sell their ideas in a business “outfit,” will this inevitably lead to a degradation of their social mission? What impact may such an attempt have on the identity of social entrepreneurs since they have been found to draw their legitimacy as activists, guardians, or even entrepreneurs primarily from a sense of social morality rather than from the business discourse directly (Parkinson & Howorth, 2008)?

### Managerial Implications

Our study offers important managerial implications. Our findings illustrate how social entrepreneurs need to be able to present themselves in different ways to different audiences. Business-oriented stakeholders expect the appropriate information on business models, customers, pricing, marketing, etc.. However, this is not necessarily the content that may be of interest for other audiences such as beneficiaries, or stakeholders from the social sector. Our study elucidates how social entrepreneurs need to meet the expectations of their audience and thus may need to communicate differently depending on the respective target group, much like a chameleon.

Impact investors need to be mindful of the importance of assessing the social impact of a venture. This is not a trivial task and they may need to draw on the expertise of professionals in the relevant subject area, for example the integration of marginalized people or the rehabilitation of juvenile offenders.

## Conclusion

Our study highlights the discursive repertoire that social entrepreneurs and their audiences draw upon in pitching situations. Our analysis of video material from live pitches shows how—paradoxically—a strong focus on the impact story may prevent social entrepreneurs from entering a shared discursive space with their business-oriented audience. The intertextual perspective allows us to better understand the power dynamics between the pitcher and their audience. We highlight how the intertextual reproduction of a larger discourse contained both enabling and constraining elements. In so doing, our study points to a crucial dilemma for social entrepreneurs: Should they intentionally “dress up” as a business if that helps to connect to gatekeepers and potential investors?

Although our study is based on rich data from interviews, observations, and videotaped pitches in three different cities in Germany and Switzerland, some dynamics may play out differently in social entrepreneurship communities in other countries. For example, pitching for resources in developing countries and among impoverished entrepreneurs may have different characteristics (Allison et al., 2015). While we acknowledge the limited generalizability of our findings, we maintain that our study sheds light on important processes that often unfold “behind closed doors.” Our unique data access enabled us to conduct real-time observations of live pitches, however, this setting also has limitations. Future research needs to explore which discourse (social or business) is more successful in convincing impact investors. Also, our research design did not allow us to control for the influence of other “variables.” For example, future research is needed to take other important aspects such as gender into account (Balachandra et al., 2019; Kanze et al., 2018) or to investigate how the audience’s perception of the moral intensity of the social problem (Smith et al., 2016) may influence their preference for a business or social discursive repertoire. Future research is also needed to illustrate how intertextuality plays out in other pitch situations and contexts. Such analysis could also elaborate on taboos (Hoon & Jacobs, 2014) or what is left unsaid by the entrepreneurs. In addition to our focus on language, it would be worthwhile to apply a visual ethnographic approach and explore how social entrepreneurs manage and manipulate visual symbols (Clarke, 2011) in pitches. Finally, if gatekeepers emphasize a business discourse to the detriment of the social in pitch situations, it would be crucial to understand what happens in the later process. For example, will such differences lead to misunderstandings and conflicts over means and ends between an investor and investee?

## Appendix

See Tables 1, 2, and 3.

**Table 1:** Overview start-ups data collection I

Nr.	Pseudonym social start-up	Location of pitch	Date of pitch
1	Multi-Generation house	Zurich	Feb 2013
2	Develop your personality	Zurich	Feb 2013
3	Green Energy	Zurich	Feb 2013
4	Urban Vegan Farming	Zurich	Feb 2013
5	Live Your Dreams	Zurich	Feb 2013
6	Gamification Sustainability Challenge	Zurich	Feb 2013
7	Mobilizing the digital youth	Zurich	Feb 2013
8	Business For Dummies	Zurich	Feb 2013
9	Local Community	Zurich	Feb 2013
10	Climate Friendly Meal	Zurich	Feb 2013
11	World Music Concerts	Zurich	Feb 2013
12	Refugee Coaching	Zurich	Feb 2013

**Table 2:** Overview start-ups data collection II

Nr.	Pseudonym social start-up	Location of pitch	Date of pitch	Status	Audience Award	Alive 2020
1	Regional food	Berlin	February 2013			x
2	Social Network	Berlin	February 2013			x
3	Soccer for everyone	Berlin	February 2013	Winner	Winner	0
4	Entertainment Education	Berlin	February 2013			x
5	Share Shop	Berlin	February 2013			x
6	Mushroom Farming	Berlin	February 2013	Winner		x
7	Sustainability Game	Berlin	February 2013	Winner		0
8	Emissionfree Food	Berlin	February 2013			0
9	Medical Backpack	Berlin	February 2013	Winner		0
10	Human Rights Online	Berlin	February 2013	Winner		0
11	Fair Handbags	Hamburg	May 2013	Winner	Winner	x
12	Solar Panels for Mosques	Hamburg	May 2013	Winner		x
13	Upcycling Design	Hamburg	May 2013			x
14	Inclusive Dentist	Hamburg	May 2013	Winner		0
15	Rural Tourism	Hamburg	May 2013	Winner		x
16	Education Holidays	Hamburg	May 2013	Winner		x
17	Clothes for cyclists	Hamburg	May 2013	Winner		0
18	Menstruation Cups	Hamburg	May 2013	Winner		x
19	Deafislam	Hamburg	May 2013	Winner		0
20	Children’s Bus	Berlin	June 2013			0
21	Secure Data	Berlin	June 2013			0
22	Women of India	Berlin	June 2013			x
23	Sustainable Finance	Berlin	June 2013	Winner		0
24	Development Internships	Berlin	June 2013			0
25	Love Clothes	Berlin	June 2013			x
26	Dreamkids	Berlin	June 2013	Winner		x
27	Sustainable Housing	Berlin	June 2013			0
28	Urban Bees	Berlin	June 2013	Winner	Winner	x
29	Urban Fitness	Hamburg	August 2013	Winner		x
30	Financial Literacy Game	Hamburg	August 2013	Winner		0
31	Games for Developmental Work	Hamburg	August 2013			0
32	Shop and Donate	Hamburg	August 2013			x
33	Death Cafe	Hamburg	August 2013	Winner		0
34	Event Agency	Hamburg	August 2013	Winner		0
35	Fairtrade Shop	Hamburg	August 2013			x
36	Blindmap	Hamburg	August 2013	Winner	Winner	0
37	Soccerfan	Hamburg	August 2013	Winner		0

**Table 3:** Key Elements of Business and Social Discourse

	Business discourse		Social discourse	
	*Ideology* Profit-maximizing strategies Emphasis on efficiency and profit		*Ideology* Shaped by values such as justice, inclusion, and responsibility Emphasis on social impact	
	Pitcher	Audience	Pitcher	Audience
Concept	*Financial viability of the business model*			
	*Revenue streams and financing options* “We want to generate sales. How do we do that? We were already doing this during the first season’s production, so to speak, mainly through advertising revenue, but also through cooperation agreements with firms.” [pitch]	*Solid business model* “Are you planning to take a loan to finance the next phase?” [audience question] “When do you expect to break even?” [audience question] “How much do you produce per month, and what is your profit margin on each product?” [Audience question]		
	*Unique Selling Proposition*		*Theory of change/social impact*	
	*Unique features of product/service* “This is a completely new product.” [pitch] “Our offer is unique.” [pitch] *Differentiation from existing offers* “How are we going to get there and what differentiates our offers from others?” [pitch]	*Market niche* “What’s your niche in the market? […] Where is the Unique Selling Proposition you are offering?”. [Audience question] “So we keep seeing projects that fill niches. Niches that I didn’t even know existed.” [interview, employee, incubator] *Differentiation from competitors* “You mentioned your competitors. What value do you add compared to other providers that are used by your target group?” [Audience question] “The market is insanely crowded. What was great yesterday is already obsolete today. The whole edutainment market, how can you differentiate yourselves?” [Audience question]	*Social problem and solution* “We want to teach teenagers financial literacy. We see a surprising lack of financial knowledge in young people [shows and explains statistics] resulting in overspending and a growing young population that is over-indebted. We provide an alternative tool, which is edutainment, education and entertainment. So we are going to teach in a way that is appropriate for our target group: a fun game which is very intuitive.” [pitch] “We have witnessed various accidents in the textile industry, for example, in Bangladesh Women work in devastating circumstances. In 2010, I was able to develop the concept of “FairFashion.” We teach women without an education how to sew. The pieces they sew are then sold in Germany and we invest the profits in local literacy projects.”[pitch]	*Reach of the offer* “Why do you support them for just 1 year? Can you leave the children on their own after that year?” [Audience question]
	*Customer*		*Beneficiary*	
	*Needs and expectations of (potential) customers* “We have conducted tests and know exactly what our customer expects from this product” [pitch]	*Number and characteristics of customers (ability to pay)* “I was impressed by the concept of Mushroom Farming, by the fact that they serve a huge customer group.” [interview, jury member] “Who is your customer?” [audience question] “So one thing that’s essential for me when I hear the pitches: do I understand the customer view?” [interview, jury member]	*In-depth knowledge of beneficiary needs* “With our experience, we have detailed knowledge of the specific needs of the target group.” [pitch] “We are looking at violent young men who love soccer [hooligans], and we want to change their behavior. They have to want to play the game, and if they are in, then we have a subtle educational effect built in.” [pitch]	*Characteristics of beneficiaries* “So I imagine I’m a young person and my future prospects are that I will be living on welfare. How do you address me?” [Audience question]
	*Pricing*		*Fair remuneration*	
	*Price of product/service* “The small bag is available from 59 Euro, the larger handbags from 149 Euro. This is not very expensive. And we have key chains for 29 Euro. This is affordable so everyone can participate.” [pitch] “We still need to determine the price. We are currently working on this.” [pitch]	*Pricing strategy and costs* “Have you already thought about the price? So how expensive is the whole thing? What about the willingness to pay? “ [Audience question] “I would have now again a question because of the financing. You said that anyone who wants to be an exhibitor should be able to set this up and staff it. Are there exhibitors who can actually afford your offer?” [Audience question]	*Comprehensive pricing model* “We pay our workers fairly, from the cotton growers to the weavers to the seamstresses, and offer them opportunities for training and regular medical checkups. Our fabrics are made from organic cotton and no toxins are released into the environment or into the clothes. We pay attention to CO2-saving transports and also work fairly and sustainably here in Germany. And that determines the price. We are a small initiative, which means that from the price you pay for a T-shirt, no profits are siphoned off for the shareholders or CEOs of a large corporation.” [pitch]	
	*Marketing*		*Reaching Beneficiaries*	
	*Presenting and advertising of the product/service* “We want to clearly position ourselves as a brand.” [pitch] “We use targeted advertising, we target the large manufacturers of sporting goods or retailers.” [pitch]	*Marketing strategy* “Congratulations on your design. Your slides and the logo look great. Would you give us more information on how your marketing works? How do you approach customers? Do you have all this as merchandising and distribute it. Is it a source of income for you?” [audience question] “Who takes care of marketing?” [pitch]	*Accessibility and inclusion of beneficiaries* “We will make sure that all those in need have access to our services.” [pitch] “We want to engage students who have had bad experiences, that is, who have been subjected to cyber bullying, who are isolated and no longer go to school.” [pitch]	*Number of users needed for (social) success* “How do you manage to reach the critical mass you need to make the concept work?” [audience question] “I was not aware of the magnitude of the problem. Do you think enough people will download the game and play?” [audience question]
	*Commercial growth*		*Scaling*	
	*Growing the business beyond the core* “You can easily transfer the App to any sport, so we can quickly reach many potential customers.” [pitch] “In addition, it may be possible to use this technology in other areas. Hence, we will apply for patent protection.” [pitch]	*Growth in revenue and sales* “You have been online with your offer for some time now. What growth do you expect in sales? What is your goal?” [audience question] “How does your franchising model exactly work?” [audience question]	*Social impact growth* “I think this idea could be implemented like this in other cities or countries.” [pitch]	
Object	*Start-up as a business*		*Start-up as a social mission driven organization*	
	*Working business model* “We have a solid business case.” [pitch]	*Emphasizing business model as essential* “Only sufficient management knowledge and a solid business model can guarantee that the start-up will survive.” [interview, jury member]	*Emphasizing social impact as key feature* “Our idea will improve the situation.” [pitch]	*Business with *“social side effects” “In a pitch, I’m blown away by ideas where I realize it reaches out to a larger audience and there are new technical possibilities with social and sustainable effects.” [interview, employee incubator]
	*Social cause framed as opportunity for business*		*Social cause framed as a problem that needs to be addressed*	*Search for simple and innovative solutions to complex social problems*
	*Present the opportunity in a social problem* “We can turn this need into a viable business model.” [pitch] “In the long term, our project will be self-financing. We will generate sufficient income to cover the costs.” [pitch] “We have this dilapidated house under monument protection and we have [social] problems in the neighborhood. We can make something out of it.” [pitch]		*Emphasizing intrinsic motivation for social change* “We had to do something about it. It can’t go on like this. If we keep consuming like we do, the world will collapse.” [pitch]	*Search for simple and innovative ideas* “But can the start-up address a problem in a particular context in a simple way—that is, offer a simple solution, the background can already be highly complex—but on the user side it must be relatively simple.” [interview, jury member]
Subject position	*Pitcher as entrepreneur*		*Pitcher as activist and changemaker*	
	*Present yourself as a business person* “This is our business idea.” [pitch] “I’m a computer scientist and have several years of experience in management and business development.” [pitch]	*Founders should be entrepreneurs* “And if they are a bit more rigid and stiff projects that are no fun, i.e., projects that are very strongly idealistically motivated, they have a hard time getting accepted. You have to see that, in principle, the market decides if they will survive.” [interview, employee incubator] “I voted against this person because I missed the entrepreneurial element in the pitch. I just didn’t see any entrepreneurial approach at all in the project.” [interview, jury member]	*Present yourself as a changemaker* “This is my calling. I want to show people a different way.” [pitch] “I work as a grief counsellor. My dream is the search that has been driving me all these years, namely, what can we do to avoid being alone in times of crisis, in times of grief? So that grief is not an individual problem, but the community becomes part of the healing journey. What do people do when they’re in mourning in other cultures? They come together and they live community. And I want to do just that in our society.” [pitch]	
	*Social entrepreneur as a person who takes risks and makes money*		*Pitcher as social innovator*	
	*Presenting yourself as a person with strong implementation skills* “We only started a few weeks ago and we have come already a long way.” [pitch] *Presenting yourself as a salesperson* “I see myself as a salesperson, a salesperson for the good cause.” [interview, presenter]	*Founders should be self-starters* “There are some people and you see them pitch and I immediately think: I trust them to make it happen.” [interview, jury member] “It is a lot about the individuals. The person is more important than the idea, I think. I need to see that the person can make her own decisions and move quickly. […] They take huge personal risks.” [interview, jury member] “What is your background? What made you want to start a business?” [audience question]	*Present yourself as a social innovator* “We really have a new approach. We want to bring young people into contact with the institutions in their city in a playful way. There was also skepticism and resistance, from the police, for example. But we were able to show them how valuable such positive contacts are.” [pitch] “Many people have called me crazy. My parents thought, they’ve invested in my education and now she’s doing something like this. [laughs] But I knew I wanted to do this and it had to work out.” [interview, founder]	*Founders should bring really new ideas to the table* “I am interested to see what’s new. If a thousand people have already tried this, then it’s not interesting.” [interview, jury member]
